# Factors associated with self- perceived speech impairment in *de novo* Parkinson’s disease: a retrospective study of an international database

**DOI:** 10.3389/fnagi.2026.1932141

**Published:** 2026-09-18

**Authors:** Zoë Thijs, Kara Bain, Matthew Dumican

**Affiliations:** 1 Department of Communication Disorders and Occupational Therapy, University of Arkansas, Fayetteville, AR, United States; 2 Department of Communication Sciences and Disorders, Molloy University, Rockville Centre, NY, United States; 3 Department of Speech, Language, and Hearing Sciences, Western Michigan University, Kalamazoo, MI, United States

**Keywords:** cognition, Parkinson’s disease, retrospective, speech, swallowing

## Abstract

**Introduction:**

People with Parkinson’s disease (PwPD) may experience speech changes early in the disease process, yet it is unclear how they perceive them. Moreover, in *de novo* PD specifically, the relationship between speech, cognition, and swallowing is not well-established. Therefore, this study aimed to determine the association between self-perceived speech impairment, clinician-perceived speech impairment, cognitive impairment, and swallowing impairment in *de novo* PD.

**Methods:**

Data were obtained from the Parkinson’s Progressive Markers Initiative. We included baseline visit data from 1072 *de novo* PwPD. An ordinal logistic regression was performed to determine what clinical measures were associated with self-perceived speech impairment.

**Results:**

The ordinal logistic regression found that self-perceived speech impairments were significantly associated with clinician-perceived speech impairment (Wald χ^2^[3] = 44.039, *p* < 0.001), self-reported swallowing impairment (Wald χ^2^[3] = 61.863, *p* < 0.001), cognitive impairment (Wald χ^2^[4] = 32.293, *p* < 0.001), and being male (Wald χ^2^[1] = 11.520, *p* < 0.001).

**Conclusion:**

This preliminary work showed potential relationships between self-perceived and clinician-perceived speech impairment, swallowing impairment, and cognitive impairment, demonstrating the multidimensional nature of self-perceived speech impairment. More research using more extensive assessment methods is needed to further elucidate these relationships in *de novo* PD.

## 1 Introduction

Parkinson’s disease (PD) is characterized primarily by motor impairments including tremor, rigidity, and bradykinesia in the extremities (Politis et al., 2010; Sveinbjornsdottir, 2016; Poewe et al., 2017), but is known to also affect speech, swallowing, and cognition (Sveinbjornsdottir, 2016). While these symptoms are well-characterized in the advanced stages of the disease, their clinical presentation is less understood in non-advanced *de novo* (medically untreated) PD. For example, changes in both speech as well as cognition are recognized as occurring in newly diagnosed people with Parkinson’s disease (PwPD) (Rusz et al., 2011, 2021; Huh et al., 2015), with acoustic speech measures sometimes being used as biomarkers for PD detection and diagnosis given how early subtle changes occur (Cao et al., 2025). However, the exact expression and progression of symptoms in these domains remain unclear, including how *de novo* PwPD perceive the above-described speech changes. To our knowledge, only one study to date has included patients classified as *de novo* in an exploration of self-perceived speech and voice. Watts and Zhang (2022) utilized the Parkinson’s Progression Markers Initiative (PPMI) data set to assess self-perceived speech and swallowing function longitudinally and identified limited impairment for patients still in the *de novo* stage.

For individuals with a longer duration PD diagnosis, speech and voice disorders are ubiquitous with about 90% of PwPD reporting speech or communication difficulties (Schalling et al., 2017). Some of the key characteristics of the speech impairment in PwPD (also known as hypokinetic dysarthria) are low intensity, monotone speech, changes in voice quality, and imprecise articulation (Sapir et al., 2008). However, studies on how PwPD perceive and report these communication changes are conflicting. An existing body of evidence has established a long-held theory that PwPD cannot adequately perceive, self-report, or self-correct speech and voice symptoms or impairment (Mollaei et al., 2019; De Groote et al., 2020; Contreras-Ruston et al., 2025). Given these potential limitations in awareness and reliability of reporting, it is theorized that measures requiring self-perception, such as patient-reported outcome measures, could present challenges in the measurement and management of these symptoms. However, numerous studies have shown that when presented with in-depth questionnaires, PwPD are able to perceive and self-report speech and voice impairment (Miller et al., 2008; van Hooren et al., 2016; Dumican and Watts, 2020; Silbergleit et al., 2021; Dumican et al., 2026) and these self-reports have also been found to align with communication partners’ external judgments of voice impairment (Parveen, 2016; Thijs et al., 2022). Consequently, it is of interest to examine the association of self-perceived speech impairment with clinician-perceived impairment. While they are different constructs, associations between self-perceived and clinician-perceived speech impairment could provide insight on whether they capture related aspects of speech impairment.

Another element that may contribute to inconsistencies regarding the ability of PwPD to perceive speech impairment is cognitive status. Prevalence of mild cognitive impairment (MCI) is estimated to be as high as 40% in recently diagnosed PD (Broeders et al., 2013; Lawson et al., 2017), and has been noted to affect cognitive functions such as attention and memory (Litvan et al., 2012; Aarsland et al., 2021), and may bear some relation to both motor and non-motor symptoms (Baiano et al., 2020). For example, Kwon et al. (2022) found that impaired performance on assessments of motor symptoms are broadly associated with early cognitive impairment. This suggests that cognitive impairment such as MCI may negatively impact PwPD’s ability to perceive and report speech impairment, which may be a motor symptom occurring simultaneously. However, few investigations involving speech and voice in PwPD directly account for cognitive impairment and studies may even exclude participants with MCI methodologically.

Finally, swallowing function is of interest when considering speech impairment. Speech and swallowing systems share neural and anatomical substrates (Ludlow, 2015). It is consequently unsurprising that in disordered populations associations between voice and swallowing measures have been described (Mira et al., 2024), including in PD (Baldanzi et al., 2025; Ko et al., 2018). In Parkinson’s disease specifically, the connection between speech and swallowing becomes even more apparent as treatment approaches that target on speech (e.g., Lee Silverman Voice Treatment) have also been demonstrated to improve swallowing function (Sharkawi et al., 2002; Miles et al., 2017; Stegemöller et al., 2017). This raises the question of whether these associations between speech and swallowing changes are already present in early stage, *de novo* Parkinson’s disease.

Consequently, the goal of the current exploratory study was to determine how client self-perception of speech impairment is associated with clinician-assessed speech impairment, cognitive impairment, and self-perceived swallowing impairment.

## 2 Materials and methods

### 2.1 Data and participants

The current project was a retrospective study of an existing, openly available database. Data used in the preparation of this article was obtained from the Parkinson’s Progression Markers Initiative (PPMI) database (www.ppmi-info.org/access-dataspecimens/download-data), RRID:SCR_006431. The PPMI database provides an open access database that collects information on the biological markers throughout the PD disease process within the context of a longitudinal, multicenter, international initiative aiming to better understand PD progression. The current project used Tier 1 PPMI Data, which are openly available. The data include extensive information on the motor and non-motor symptoms of PD across hundreds of PwPD, prodromal patients, and healthy controls. To create the data set, the curated dataset available within the PPMI data was downloaded, and responses to the selected Movement Disorder Society-sponsored Unified Parkinson’s Disease Rating Scale (MDS-UPDRS) items (see below) were manually linked and merged with the curated dataset using participant numbers and event IDs. The resulting data set was used for all analyses. Unless otherwise stated, the original coding of the variables of the PPMI was preserved.

The current study screened data from a total of 16,126 cases of PwPD, and only included data from participants with sporadic PD at their baseline visit who were not receiving pharmaceutical treatment for PD and had not received deep brain stimulation (DBS). Treatment status was determined from the PDTRTMNT variable available in the curated dataset. The before-mentioned criteria were selected to align with the purposes of this study to examine a newly diagnosed, non-advanced sample of PwPD. Participants with missing data for independent and dependent variables (described below) were excluded listwise. A flow chart describing participant selection is available in Figure 1. The following demographic information was included in the study: age, sex, race, educational years, disease duration, MDS-UPDRS subpart scores and total scores. A complete list with specific variables used can be found in Table 1.

**FIGURE 1:**
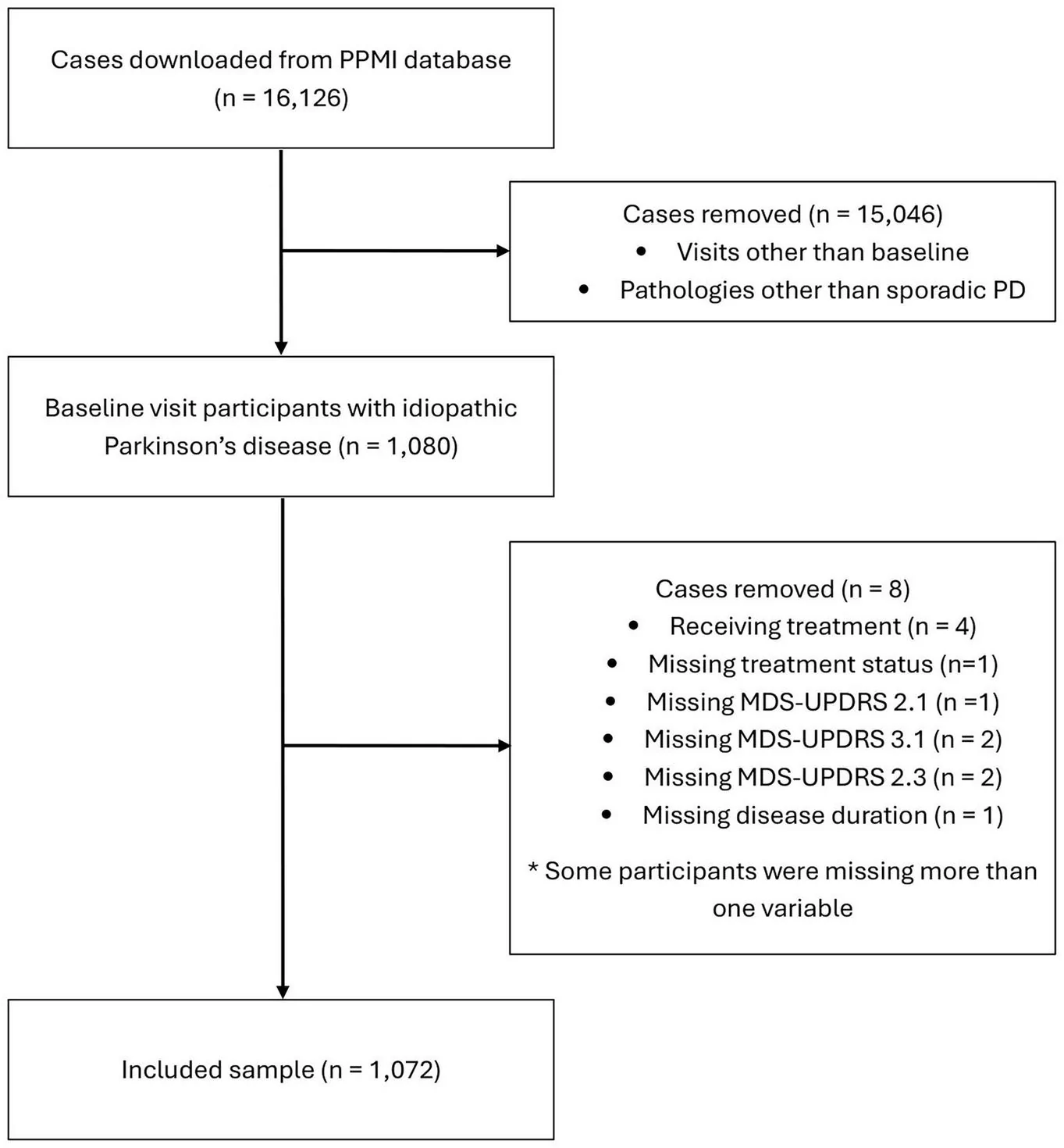
Flowchart describing participant selection from the PPMI database.

**TABLE 1:** Participant characteristics of the PwPD (*n* = 1,072) included in the sample.

Variable	Category	Mean (SD)
Age (years)		63.25 (9.46)
Educational years (years)		15.99 (2.73)
Disease duration (years)		0.73 (0.60)
		**Frequency (percentage)**
Sex	Male	704 (65.7%)
	Female	368 (34.3%)
Race	White	991 (92.4%)
	Black	21 (2.0%)
	Asian	18 (1.7%)
	Other	35 (3.3%)
	Unknown	7 (0.7%)
		**Mean (SD)** **Median (IQR)**
MDS-UPDRS Part I		6.28 (4.47)
		5.00 (3.00–9.00)
MDS-UPDRS Part II		6.36 (4.49)
		5.00 (3.00–9.00)
MDS-UPDRS Part III		22.39 (9.66)
		21.00 (15.00–28.00)
MDS-UPDRS Total		35.03 (14.46)
		33.00 (24.00–43.00)

IQR, interquartile range for the median; SD, standard deviation to the mean.

### 2.2 Measures

An ordinal logistic regression analysis was performed to assess which clinical and disease-specific measures were associated with self-perceived speech impairment. The dependent variable of interest was the response to question 2.1 (“Speech”) from Part II of the MDS-UPDRS, the patient questionnaire, and is available as NP2SPCH in the PPMI MDS-UPDRS Part II dataset. The question asks the participant to rate any experienced speech problems on a scale from 0 to 4, with 0 representing “normal” function and 4 representing “severe” impairment, similar to other MDS-UPDRS questions.

For the independent variables and primary variables of interest, we included a measure of clinician assessment of speech via Question 3.1 of the MDS-UPDRS, “Speech” (part of the motor examination, NP3SPCH variable in the PPMI MDS-UPDRS Part III dataset). While the anchors for this question and question 2.1 are different, both questions use intelligibility (i.e., ability to be understood or understanding the participant, for question 2.1 and 3.1, respectively) as a central concept. Examining the association between patient- and clinician-perceived speech impairment may provide insight into how these ratings are related to one another early in the disease process in a large sample size. A second independent variable included was self-perceived swallowing impairment via Question 2.3 of the MDS-UPDRS, “Chewing and Swallowing” (part of the patient questionnaire, variable NP2SWAL in the PPMI MDS-UPDRS Part II dataset), as relationships between speech and swallowing in people with Parkinson’s disease have been previously established (see above). Cognitive impairment as measured in Question 1.1 of the MDS-UPDRS, “Cognitive Impairment” (NP1COG variable within the PPMI MDS-UPDRS Part I dataset) was also included in the independent variables, given the above-discussed potential relationship between cognition and self-reports. This question is part of the MDS-UPDRS Part I, for which the clinician interviews the patient and/or caregiver about the non-motor symptoms of PD. Following the standardized PPMI and MDS-UPDRS protocols, all MDS-UPDRS questions were completed by consistent raters, with different designated raters at each site, who were not blinded for the results on the other MDS-UPDRS sections for clinician-rated section of Part I and Part III of the MDS-UPDRS. This common method could lead to bias, as discussed in the limitations. Part II of the MDS-UPDRS is part of the patient questionnaire and was filled out by the participant with or without caregiver help.

To control for aspects of disease-related variability, we included additional covariates in the analysis. Biological sex and age of the participant, as well as disease duration were added as independent variables, given that these factors can impact Parkinson’s disease progression and symptom manifestation (Picillo et al., 2021; Virameteekul et al., 2021; Raket et al., 2022; Hanff et al., 2025). This data was all available in the curated data set as sex, age_at_visit and duration_yrs variables, respectively.

### 2.3 Statistical analysis

First, all data were analyzed descriptively. Averages and standard deviations were calculated for continuous variables, frequencies were reported for nominal variables, and mean, median, and interquartile range (IQR) were calculated for ordinal data.

Next, inferential statistical analyses were performed using an alpha level of 0.05. A cumulative odds ordinal logistic regression was planned in IBM SPSS Statistics (Version 31) using the dependent and independent variables listed above. Preliminary tests confirmed that assumptions of the statistical test were met, including tolerance (>0.9), VIF (<1.10), and the Test of Parallel Lines (χ^2^[26] = 26.023, *p* = 0.462) showing proportional odds. Consequently, the ordinal regression was completed.

Additionally, a sensitivity analysis using a generalized linear mixed model using a multinomial distribution and cumulative logit link function was performed to assess potential clustering by site where the data was collected. The same dependent and independent variables were used, with a random intercept for site (SITE variable within the curated data set) specified using variance components covariance structure. Because the site-level analysis suggested minimal clustering by site (see Section “3 Results” below), the primary ordinal logistic regression was retained as the main analysis. Accordingly, only the key findings from the mixed-model sensitivity analysis were reported below.

## 3 Results

### 3.1 Participants

As noted previously, the data records for 16,126 cases were screened for inclusion in the analysis. The final analysis included 1072 PwPD, after excluding follow-up visits, non-sporadic PD, and incomplete records. Participant characteristics are described in Table 1. The average age across the included sample was 63.25 years, with a PD disease duration of less than 1 year on average. Descriptive statistics for the other variables included in the ordinal regression can be found in Table 2.

**TABLE 2:** Descriptive statistics of the categorical variables included in the ordinal logistic regression.

	Median (IQR)	Frequency (percentage) [*n* = 1,072]				
Variable		Normal	Slight	Mild	Moderate	Severe
Self-perceived speech impairment (MDS-UPDRS 2.1)	0.00 (0.00–1.00)	681 (63.5%)	265 (24.7%)	112 (10.4%)	14 (1.3%)	0 (0.0%)
Clinician-perceived speech impairment (MDS-UPDRS 3.1)	0.00 (0.00–0.00)	592 (55.2%)	432 (40.3%)	46 (4.3%)	2 (0.2%)	0 (0.0%)
Self-perceived swallowing impairment (MDS-UPDRS 2.3)	0.00 (0.00–1.00)	891 (83.1%)	163 (15.2%)	7 (0.7%)	11 (1.0%)	0 (0.0%)
Cognitive impairment (MDS-UPDRS 1.1)	0.00 (0.00–1.00)	772 (72%)	256 (23.9%)	33 (3.1%)	10 (0.9%)	1 (0.1%)

IQR, interquartile range for the median.

### 3.2 Ordinal logistic regression

A cumulative odds ordinal logistic regression was performed to determine the effect of clinician-perceived speech impairment (MDS-UPDRS 3.1), self-perceived swallowing impairment (MDS-UPDRS 2.3), cognitive impairment (MDS-UPDRS 1.1), age, sex, and disease duration on self-perceived speech impairment (MDS-UPDRS 2.1). The final model statistically significantly predicted the dependent variable over the intercept only model (χ^2^[13] = 183.120, *p* < 0.001). The full results are available in Table 3.

**TABLE 3:** Summary of ordinal logistic regression analysis of self-perceived speech impairment (MDS-UPDRS 2.1).

Variable	B	SE	Wald χ ^2^	*P*	e^B^	95% CI for e^B^
Clinician-perceived speech impairment (MDS-UPDRS 3.1)						
3 - Moderate	2.373	1.3620	3.035	0.081	10.727	0.743–154.823
2 - Mild	1.511	0.2973	25.834	<0.001*	4.532	2.531–8.117
1 - Slight	0.732	0.1385	27.921	<0.001*	2.079	1.585–2.727
0 - Normal						
Self-perceived swallowing impairment (MDS-UPDRS 2.3)						
3 - Moderate	2.238	0.5728	15.261	<0.001*	9.373	3.050–28.805
2 - Mild	0.750	0.7328	1.046	0.306	2.116	0.503–8.898
1 - Slight	1.189	0.1676	50.325	<0.001*	3.285	2.365–4.562
0 - Normal						
Cognitive impairment (MDS-UPDRS 1.1)						
4 - Severe	2.605	1.8420	2.000	0.157	13.533	0.366–500.370
3 - Moderate	1.784	0.6102	8.544	0.003*	5.951	1.800–19.679
2 - Mild	1.016	0.3421	8.819	0.003*	2.762	1.413–5.400
1 - Slight	0.639	0.1483	18.550	<0.001*	1.894	1.416–2.533
0 - Normal						
Sex						
Male	0.493	0.1453	11.520	<0.001*	1.637	1.232–2.176
Female						
Age	0.004	0.0072	0.244	0.622	1.004	0.989–1.018
Duration	0.072	0.1085	0.446	0.504	1.075	0.869–1.330

*N* = 1,072. B, unstandardized regression coefficient; SE, standard error; OR, odds ratio; CI, confidence interval. Reference categories are “0 - Normal” for clinician-perceived speech, self-perceived swallowing, cognitive impairments, and “female” for Sex. *Statistically significant at the 0.05 level.

Clinician-perceived levels of speech impairment were significantly associated with self-perceived speech impairment (Wald χ^2^[3] = 44.039, *p* < 0.001). Compared to the normal group for the clinician-perceived speech impairment measure, the slight group had 2 times higher odds of rating their own speech as more impaired (OR = 2.079, 95% CI [1.585–2.727], *p* < 0.001), and the mild clinician-perceived speech impairment group odds were 4.5 times higher (OR = 4.532, 95% CI [2.531–8.117], *p* < 0.001).

Similarly, self-reported swallowing impairment was significantly associated with self-perceived speech impairment (Wald χ^2^[3] = 61.863, *p* < 0.001). Compared to participants with normal self-rated swallowing function, participants with slight self-perceived swallowing problems were three times more likely to rate their speech as more impaired (OR = 3.285, 95% CI [2.365–4.562], *p* < 0.001), with even higher odds for those who rated their swallow as moderately impacted (OR = 9.373, 95% CI [3.050–28.805], *p* < 0.001).

Additionally, cognitive impairment was significantly associated with self-perceived speech impairment (Wald χ^2^[4] = 32.293, *p* < 0.001). The odds of rating their own speech as impaired increased across the slight (OR = 1.894, 95% CI [1.416–2.533], *p* < 0.001), mild (OR = 2.762, 95% CI [1.413–5.400], *p* = 0.003), and moderate (OR = 5.951, 95% CI [1.800–19.679], *p* = 0.003) severities of cognitive impairment, compared to participants with cognitive impairment rated as normal.

However, because several of the more impaired speech, swallowing and cognitive categories contained very few participants (see Table 2), these estimates need to be interpreted cautiously.

Finally, sex was a significant predictor in the model (Wald χ^2^[1] = 11.520, *p* < 0.001). Male participants had higher odds of rating their speech as more impaired compared to female participants (OR = 1.637, 95% CI [1.232–2.176], *p* < 0.001).

### 3.3 Sensitivity analysis for site

The data in the current sample was collected on 51 sites. The number of participants per site ranged from 1 participant (0.1% of total sample) to 94 participants (8.8% of sample) (median = 14, interquartile range = 10–28.5). As described above, potential site clustering was assessed with a general linear mixed model, which was statistically significant (*F*[13,1056] = 13.040, *p* < 0.001). Consistent with the primary ordinal logistic regression, clinician-perceived levels of speech impairment (*F*[3,1056] = 15.332, *p* < 0.001), self-perceived swallowing impairment (*F*[3,1056] = 20.703, *p* < 0.001), cognitive impairment (*F*[4,1056] = 7.368, *p* < 0.001), and sex (*F*[1,1056] = 11.004, *p* < 0.001) showed statistically significant associations, while age at visit and disease duration did not. The site level variance was small (variance = 0.099, SE = 0.065, *p* = 0.126), indicating minimal site clustering. Consequently, the original ordinal logistic regression (see above) was retained as the primary analysis.

## 4 Discussion

The current study aimed to investigate how clinician-perceived speech impairment, self-perceived swallowing impairment, and cognitive impairment relate to self-perceived speech impairment in *de novo*, untreated PD. Overall, we found significant associations among these variables. Generally speaking, greater levels of clinician-rated speech, self-perceived swallowing impairment, and cognitive impairment were associated with higher self-perceived ratings of speech impairment. In addition, men were more likely to report greater speech impairment. Given the paucity of information on the relationships between self-perception and clinician-assessment of speech function in early-stage *de novo* PD, this study provides important, emerging data on the topic in a large sample of *de novo* PwPD.

### 4.1 Self-perceived and clinician-perceived speech impairment

The current study found that self-perceived speech impairment, as measured by MDS-UPDRS 2.1, and clinician-perceived speech impairment, as measured by MDS-UPDRS 3.1, were significantly associated, with higher scores on one co-occurring with higher scores on the other. These MDS-UPDRS questions both use a five-point severity scale (normal–slight–mild–moderate–severe), but differ in their rating anchors. While both scales refer to intelligibility as a key component, they do so in different contexts: speech being understood by others (question 2.1) and the clinician understanding the patient’s speech (question 3.1), respectively. It is important to note that these questions provide limited information on “objective” speech impairment compared to a formal assessment, and may be more suited to screening. Nevertheless, finding an association between self-perceived and clinician-perceived speech impairment on screening questions provides some initial insight. Relationships between client and clinician or other outside ratings (e.g., communication partner ratings) have been described previously in more advanced stages of PD. For example, Parveen (2016) found no group differences between motor ratings of patients, their communication partner, and trained experts. Similarly, for different tools assessing speech, studies found no group differences between PwPD and their communication partner (Miller et al., 2008; Dykstra et al., 2015; Parveen and Goberman, 2017; Thijs et al., 2022) and/or high levels of agreement between the two groups (Parveen and Goberman, 2017; Thijs et al., 2022). Still, other studies report differences between client, communication partner, and trained rater judgment (Donovan et al., 2008; Parveen and Goberman, 2016). The current study could represent an expansion of previously limited knowledge on these relationships specifically in *de novo* PwPD supporting associations between self- and clinician-perceived speech impairment. However, more research is needed in *de novo* Parkinson’s disease to further explore potential relationships between client and clinician ratings of speech in greater depth using validated and clinically relevant measures of speech impairment.

### 4.2 Self-perceived speech and self-perceived swallowing impairment

Similarly, a relationship was found between self-reported speech impairment (MDS-UPDRS 2.1) and swallowing impairment (MDS-UPDRS 2.3), with higher levels of self-rated swallowing impairment being associated with higher levels of speech impairment. While needing to be interpreted with caution given the limited information incorporated in this question, a potential association between speech and swallowing even in early PD is clinically important. Other studies, in later stage and treated PD, have also described connections between speech and swallowing using more robust measures. For example, Dumican and Watts (2020) found that self-perceived vocal function was associated with self-perceived swallowing function. van Hooren et al. (2016) also found significant correlations between participant reports on voice and swallowing-related quality of life questionnaires. These findings could provide a preliminary indication that even in *de novo*, early-stage PD speech and swallowing function are related, though further research with more extensive assessment is required. Clinically, these results may mean that when impairment in one is reported on a screening tool, the other function should also be considered for screening and more comprehensive assessment.

### 4.3 Self-perceived speech and cognitive impairment

A third association was found between self-perceived speech (MDS-UPDRS 2.1) and cognitive impairment (MDS-UPDRS 1.1). As with the previously discussed results from this study, it is necessary to use caution in interpreting this relationship between self-perceived speech and cognitive impairment in *de novo* Parkinson’s disease, given the limited information provided by these test items. However, it is not a novel phenomenon to find relationships between speech presentation and cognitive function in PD. For example, Polychronis et al. (2019) found that PwPD with speech difficulties also showed increased cognitive decline at year three follow up using the PPMI database. Gago et al. (2009) and Rektorova et al. (2016) found similar relationships, where greater degree of speech impairment in PwPD predicted poorer cognitive performance on a variety of tests. When examining the concept of speech in functional communication abilities, a systematic review found that increased incidence of diagnosed cognitive impairment led to increased difficulties with everyday communication (Barnish et al., 2016). Potential explanations for this phenomenon could be that speech and cognition deteriorate in tandem, or that the MDS-UPDRS questions on cognition and self-perceived speech address similar underlying constructs. However, the potential link between cognitive function and self-perceived speech highlights the importance of speech-language pathologists screening for not just speech and swallowing, but also cognition in PwPD.

### 4.4 Self-perceived speech and sex

The effect of sex on speech in PwPD has recently received more attention. In established PD, some studies found male patients to present with increased speech and voice difficulties (Skodda et al., 2011; Russillo et al., 2022; Santos-García et al., 2023), while others found females to have changes in prosody and voice quality (Hertrich and Ackermann, 1995; Skodda et al., 2011), with another study not finding any sex differences for most speech parameters (Houle et al., 2024). In *de novo* PD, Rusz et al. (2022) echoed these last findings with no gender-specific differences in acoustic voice quality. In contrast to the Rusz et al. (2022) study, we found that being male was associated with reports of greater speech impairment in our *de novo* PD sample. However, it is important to note that subjective self-perception of speech and objective acoustic analysis of speech represent two very different constructs. Another consideration may be the inherently unbalanced distribution of each sex in the available dataset. The sample utilized for this study consisted of over 65% male participants, which is not uncommon in PD-related research. Studies examining a more even sex distribution regarding these specific variables may be warranted to confirm the contribution of sex to the variables of interest. Given that studies have shown sex-specific differences in the progression of Parkinson’s disease (Hanff et al., 2025) and the contradictory preliminary evidence for sex-based differences in speech discussed above, substantially more research is needed on how these sex-based differences may express themselves in speech, especially given that sex-specific speech differences may be difficult to differentiate from sex-specific disease impacts.

### 4.5 Limitations

As with every study, the current study also presents with limitations. First, given the robust sample size of the PPMI database, missing data were deleted list-wise from the analysis. It is possible that, in doing so, the characteristics of the included sample are different than that of the PPMI database as a whole. Consequently, the current results can only be generalized to populations with PD that present with similar characteristics as those in our sample.

Moreover, the use of MDS-UPDRS question responses provides limited information on speech, swallowing, and cognition, as the MDS-UPDRS questions consist of ordinal data with only 5 levels at one point in time. While this data on the MDS-UPDRS can serve as a helpful screening tool for clinicians working with PwPD, it does not replace a full assessment describing speech, swallowing, and cognitive function. Future, prospective studies can consider doing more extensive assessments of speech, swallowing, and cognitive function in large cohorts of early-stage, non-medicated PwPD. It is also important to note that, in this *de novo* sample of people with Parkinson’s disease, only a small proportion of participants received ratings in the mild, moderate, or severe categories on the MDS-UPDRS items included in the analysis. Though this is clinically reasonable in a *de novo*, early-stage PD population, this means that several categories within the MDS-UPDRS items contained a small number of participants, resulting in wide confidence intervals and less precise regression estimates. While these categories were maintained to preserve clinically relevant distinctions, the associated results should be interpreted cautiously. Additionally, given that most of the data included in this study were derived from the same tool (i.e., the MDS-UPDRS) that was administered by a single non-blinded assessor and combined with patient report, the found associations, may be due to the use of a common method. Consequently, the present results should be interpreted carefully and be treated as preliminary data only.

Finally, given the exploratory nature of the study, not all potentially confounding patient-related factors were included and controlled for in the statistical analyses. Patient-related factors such as educational level, depression, anxiety, and a myriad of additional patient-centered factors may influence patient self-report, and in turn the findings of the study. These variables were not included to maintain a parsimonious and interpretable statistical model, but could have nevertheless contributed to variability in the study results.

## 5 Conclusion

In conclusion, this study provides an initial and preliminary analysis of the association between self-perceived speech impairment, clinician-perceived speech impairment, self-perceived swallowing impairment, and cognitive impairment in a large population of early-stage, untreated PwPD. Greater levels of clinician-perceived speech impairment, self-perceived swallowing impairment, and cognitive impairment were associated with increased odds of self-reporting speech impairment. Moreover, male participants were more likely to report greater speech impairments. While these preliminary findings need to be interpreted with caution, as they were derived from responses to screening questions, they provide initial insight in the multifactorial nature of self-perceived speech impairment in *de novo* PD. This highlights the need to consider speech, swallowing, and cognition together, including the subjective experience of the individuals in these domains, when screening and evaluating PwPD. More research is needed to further describe these relationships in greater detail.

## Data Availability

Data used in the preparation of this article was obtained on 2025-07-22 from the Parkinson’s Progression Markers Initiative (PPMI) database (www.ppmi-info.org/access-dataspecimens/download-data), RRID:SCR_006431. For up-to-date information on the study, visit www.ppmi-info.org.

## References

[B1] Aarsland D. Batzu L. Halliday G. M. Geurtsen G. J. Ballard C. Ray Chaudhuri K. et al. (2021). Parkinson disease-associated cognitive impairment. *Nat. Rev. Dis. Primers* 7:47. 10.1038/s41572-021-00280-3 34210995

[B2] Baiano C. Barone P. Trojano L. Santangelo G. (2020). Prevalence and clinical aspects of mild cognitive impairment in Parkinson’s disease: A meta-analysis. *Mov. Disord.* 35, 45–54. 10.1002/mds.27902 31743500

[B3] Baldanzi C. Crispiatico V. Fusari G. Saibene F. L. Arcuri P. Meloni M. et al. (2025). Correlation between voice intensity and swallowing function in subjects with Parkinson’s disease. *Neurol. Sci.* 46, 713–721. 10.1007/s10072-024-07782-4 39375256

[B4] Barnish M. S. Whibley D. Horton S. M. C. Butterfint Z. R. Deane K. H. O. (2016). Roles of cognitive status and intelligibility in everyday communication in people with Parkinson’s disease: A systematic review. *J. Parkinson’s Dis.* 6, 453–462. 10.3233/JPD-150757 27003782

[B5] Broeders M. de Bie R. M. A. Velseboer D. C. Speelman J. D. Muslimovic D. Schmand B. (2013). Evolution of mild cognitive impairment in Parkinson disease. *Neurology* 81, 346–352. 10.1212/WNL.0b013e31829c5c86 23794682

[B6] Cao F. Vogel A. P. Gharahkhani P. Renteria M. E. (2025). Speech and language biomarkers for Parkinson’s disease prediction, early diagnosis and progression. *NPJ Parkinsons Dis.* 11:57. 10.1038/s41531-025-00913-4 40128529 PMC11933288

[B7] Contreras-Ruston F. Arriagada-Concha H. Lagos-Villaseca A. Rojas S. Napolitano C. Kotz S. A. et al. (2025). Investigating how age affects self-perception and voice awareness in Parkinson’s disease. *J. Voice* [Epub ahead of print]. 10.1016/j.jvoice.2025.02.032 40069018

[B8] De Groote E. De Keyser K. Bockstael A. Botteldooren D. Santens P. De Letter M. (2020). Central auditory processing in parkinsonian disorders: A systematic review. *Neurosci. Biobehav. Rev.* 113, 111–132. 10.1016/j.neubiorev.2020.03.001 32145223

[B9] Donovan N. J. Kendall D. L. Young M. E. Rosenbek J. C. (2008). The communicative effectiveness survey: Preliminary evidence of construct validity. *Am. J. Speech-Lang. Pathol.* 17, 335–347. 18957572 10.1044/1058-0360(2008/07-0010)

[B10] Dumican M. Watts C. (2020). Self-perceptions of speech, voice, and swallowing in motor phenotypes of Parkinson’s disease. *Clin. Park. Relat. Disord.* 3:100074. 10.1016/j.prdoa.2020.100074 34316653 PMC8298760

[B11] Dumican M. Harper K. Malczewski A. (2026). Self-Reported voice and swallow questionnaires’ alignment with Unified Parkinson’s Disease Rating Scale questions: A preliminary study. *J. Voice.* 40:1240. 10.1016/j.jvoice.2024.03.022 38614893

[B12] Dykstra A. D. Adams S. G. Jog M. (2015). Examining the relationship between speech intensity and self-rated communicative effectiveness in individuals with Parkinson’s disease and hypophonia. *J. Commun. Disord.* 56, 103–112. 10.1016/j.jcomdis.2015.06.012 26188950

[B13] Gago M. F. Garrett M. C. Fonseca M. R. Rosas M. J. Simões M. F. Vieira S. et al. (2009). How do cognitive and axial motor signs correlate in Parkinson’s disease? A 6-year prospective study. *J. Neurol.* 256, 1655–1662. 10.1007/s00415-009-5174-7 19471849

[B14] Hanff A.-M. McCrum C. Rauschenberger A. Aguayo G. A. Pauly C. Jónsdóttir S. R. et al. (2025). Sex-specific progression of Parkinson’s disease: A longitudinal mixed-models analysis. *J. Parkinsons Dis.* 15, 805–818. 10.1177/1877718X251339201 40388933 PMC13347478

[B15] Hertrich I. Ackermann H. (1995). Gender-Specific vocal dysfunctions in Parkinson’s disease: Electroglottographic and acoustic analyses. *Ann. Otol. Rhinol. Laryngol.* 104, 197–202. 10.1177/000348949510400304 7872602

[B16] Houle N. Feaster T. Mira A. Meeks K. Stepp C. E. (2024). Sex differences in the speech of persons with and without Parkinson’s disease. *Am. J. Speech-Lang. Pathol.* 33, 96–116. 10.1044/2023_AJSLP-22-00350 37889201 PMC11000784

[B17] Huh Y. E. Park J. Suh M. K. Lee S. E. Kim J. Jeong Y. et al. (2015). Differences in early speech patterns between Parkinson variant of multiple system atrophy and Parkinson’s disease. *Brain Lang.* 147, 14–20. 10.1016/j.bandl.2015.04.007 25997172

[B18] Ko E. J. Chae M. Cho S.-R. (2018). Relationship between swallowing function and maximum phonation time in patients with parkinsonism. *Ann. Rehabil. Med.* 42, 425–432. 10.5535/arm.2018.42.3.425 29961740 PMC6058589

[B19] Kwon K.-Y. Park S. Kim R. O. Lee E. J. Lee M. (2022). Associations of cognitive dysfunction with motor and non-motor symptoms in patients with de novo Parkinson’s disease. *Sci. Rep.* 12:11461. 10.1038/s41598-022-15630-8 35794147 PMC9259652

[B20] Lawson R. A. Yarnall A. J. Duncan G. W. Breen D. P. Khoo T. K. Williams-Gray C. H. et al. (2017). Stability of mild cognitive impairment in newly diagnosed Parkinson’s disease. *J. Neurol. Neurosurg. Psychiatry* 88, 648–652. 10.1136/jnnp-2016-315099 28250029 PMC5537517

[B21] Litvan I. Goldman J. G. Tröster A. I. Schmand B. A. Weintraub D. Petersen R. C. et al. (2012). Diagnostic criteria for mild cognitive impairment in Parkinson’s disease: Movement disorder society task force guidelines. *Mov. Disord.* 27, 349–356. 10.1002/mds.24893 22275317 PMC3641655

[B22] Ludlow C. L. (2015). Central nervous system control of voice and swallowing. *J. Clin. Neurophsiol.* 32, 294–303. 10.1097/WNP.0000000000000186 PMC4526113 26241238

[B23] Miles A. Jardine M. Johnston F. de Lisle M. Friary P. Allen J. (2017). Effect of Lee Silverman Voice Treatment (LSVT LOUD^®^) on swallowing and cough in Parkinson’s disease: A pilot study. *J. Neurol. Sci.* 383, 180–187. 10.1016/j.jns.2017.11.015 29246611

[B24] Miller N. Noble E. Jones D. Allcock L. Burn D. J. (2008). How do I sound to me? Perceived changes in communication in Parkinson’s disease. *Clin. Rehabil.* 22, 14–22. 10.1177/0269215507079096 18089662

[B25] Mira A. S. Goldsberry L. J. Previtera M. J. Simmons A. K. McKenna V. S. (2024). A scoping review on the intersection between voice and swallowing measures in healthy and disordered populations. *Am. J. Speech-Lang. Pathol.* 33, 3082–3099. 10.1044/2024_AJSLP-24-00025 39196816

[B26] Mollaei F. Shiller D. M. Baum S. R. Gracco V. L. (2019). The relationship between speech perceptual discrimination and speech production in Parkinson’s disease. *J. Speech Lang. Hear. Res.* 62, 4256–4268. 10.1044/2019_JSLHR-S-18-0425 31738857

[B27] Parveen S. (2016). Comparison of self and proxy ratings for motor performance of individuals with Parkinson disease. *Brain Cogn.* 103, 62–69. 10.1016/J.BANDC.2016.01.006 26837016

[B28] Parveen S. Goberman A. M. (2017). Comparison of self and proxy ratings for voice handicap index and motor-related quality-of-life of individuals with Parkinson’s disease. *Intern. J. Speech-Lang. Pathol.* 19, 174–183. 10.3109/17549507.2016.1167242 27146109

[B29] Parveen S. Goberman A. M. (2016). Comparison of Parkinsonian speech ratings by individuals with Parkinson disease, communication partners, and a trained rater. *Speech Lang. Hear.* 25, 138–152. 10.1080/2050571X.2020.1826108

[B30] Picillo M. LaFontant D.-E. Bressman S. Caspell-Garcia C. Coffey C. Cho H. R. et al. (2021). Sex-Related longitudinal change of motor, non-motor, and biological features in early Parkinson’s disease. *J. Parkinson’s Dis.* 12, 421–436. 10.3233/JPD-212892 34744052 PMC8842783

[B31] Poewe W. Seppi K. Tanner C. M. Halliday G. M. Brundin P. Volkmann J. et al. (2017). Parkinson disease. *Nat. Rev. Dis. Primers* 3:17013. 10.1038/nrdp.2017.13 28332488

[B32] Politis M. Wu K. Molloy S. Bain P. G. Chaudhuri K. R. Piccini P. (2010). Parkinson’s disease symptoms: The patient’s perspective. *Mov. Disord.* 25, 1646–1651. 10.1002/mds.23135 20629164

[B33] Polychronis S. Niccolini F. Pagano G. Yousaf T. Politis M. (2019). Speech difficulties in early de novo patients with Parkinson’s disease. *Parkinson. Related Disord.* 64, 256–261. 10.1016/j.parkreldis.2019.04.026 31078401

[B34] Raket L. L. Oudin, Åström D. Norlin J. M. Kellerborg K. Martinez-Martin P. et al. (2022). Impact of age at onset on symptom profiles, treatment characteristics and health-related quality of life in Parkinson’s disease. *Sci. Rep.* 12:526. 10.1038/s41598-021-04356-8 35017548 PMC8752787

[B35] Rektorova I. Mekyska J. Janousova E. Kostalova M. Eliasova I. Mrackova M. et al. (2016). Speech prosody impairment predicts cognitive decline in Parkinson’s disease. *Parkinson. Relat. Disord.* 29, 90–95. 10.1016/j.parkreldis.2016.05.018 27237105

[B36] Russillo M. C. Andreozzi V. Erro R. Picillo M. Amboni M. Cuoco S. et al. (2022). Sex differences in Parkinson’s disease: From bench to bedside. *Brain Sci.* 12:917. 10.3390/brainsci12070917 35884724 PMC9313069

[B37] Rusz J. Cmejla R. Ruzickova H. Klempir J. Majerova V. Picmausova J. et al. (2011). “Acoustic Markers of Speech Degradation in Early Untreated Parkinson’s Disease,” in *Proceedings of Forum Acusticum 2011: 27 June - 01 July*, (Aalborg), 2725–2730.

[B38] Rusz J. Tykalová T. Novotný M. Zogala D. Růžička E. Dušek P. (2022). Automated speech analysis in early untreated Parkinson’s disease: Relation to gender and dopaminergic transporter imaging. *Eur. J. Neurol.* 29, 81–90. 10.1111/ene.15099 34498329

[B39] Rusz J. Tykalova T. Novotny M. Zogala D. Sonka K. Ruzicka E. et al. (2021). Defining speech subtypes in de novo Parkinson disease: Response to long-term levodopa therapy. *Neurology* 97, e2124–e2135. 10.1212/WNL.0000000000012878 34607922

[B40] Santos-García D. Laguna A. Hernández-Vara J. de Deus Fonticoba T. Cores Bartolomé C. Feal Painceiras M. J. et al. (2023). Sex differences in motor and non-motor symptoms among spanish patients with Parkinson’s disease. *J. Clin. Med.* 12:1329. 10.3390/jcm12041329 36835866 PMC9960095

[B41] Sapir S. Ramig L. O. Fox C. (2008). Speech and swallowing disorders in Parkinson disease. *Curr. Opin. Otolaryngol. Head Neck Surg.* 16, 205–210. 10.1097/MOO.0b013e3282febd3a 18475072

[B42] Schalling E. Johansson K. Hartelius L. (2017). Speech and communication changes reported by people with Parkinson’s disease. *Folia Phoniatrica Logopaedica* 69, 131–141. 10.1159/000479927 29346787

[B43] Sharkawi A. E. Ramig L. Logemann J. A. Pauloski B. R. Rademaker A. W. Smith C. H. et al. (2002). Swallowing and voice effects of Lee Silverman Voice Treatment (LSVT^®^): A pilot study. *J. Neurol. Neurosurg. Psychiatry* 72, 31–36. 10.1136/jnnp.72.1.31 11784821 PMC1737706

[B44] Silbergleit A. K. Schultz L. Hamilton K. LeWitt P. A. Sidiropoulos C. (2021). Self-Perception of voice and swallowing handicap in Parkinson’s disease. *J. Parkinsons Dis.* 11, 2027–2034. 10.3233/JPD-212621 34366369

[B45] Skodda S. Visser W. Schlegel U. (2011). Gender-Related patterns of dysprosody in parkinson disease and correlation between speech variables and motor symptoms. *J. Voice* 25, 76–82. 10.1016/j.jvoice.2009.07.005 20381998

[B46] Stegemöller E. L. Hibbing P. Radig H. Wingate J. (2017). Therapeutic singing as an early intervention for swallowing in persons with Parkinson’s disease. *Compl. Ther. Med.* 31, 127–133. 10.1016/j.ctim.2017.03.002 28434465

[B47] Sveinbjornsdottir S. (2016). The clinical symptoms of Parkinson’s disease. *J. Neurochem.* 139, 318–324. 10.1111/jnc.13691 27401947

[B48] Thijs Z. Zhang Y. Van Lierde K. Vanryckeghem M. Watts C. R. (2022). Partner perception of affective, behavioral, and cognitive reactions to voice use in people with Parkinson’s disease. *Clin. Parkinson. Related Disord.* 7:100152. 10.1016/j.prdoa.2022.100152 35860426 PMC9289734

[B49] van Hooren M. R. A. Baijens L. W. J. Vos R. Pilz W. Kuijpers L. M. F. Kremer B. et al. (2016). Voice- and swallow-related quality of life in idiopathic Parkinson’s disease. *Laryngoscope* 126, 408–414. 10.1002/lary.25481 26153234

[B50] Virameteekul S. Phokaewvarangkul O. Bhidayasiri R. (2021). Profiling the most elderly parkinson’s disease patients: Does age or disease duration matter? *PLoS One* 16:e0261302. 10.1371/journal.pone.0261302 34937068 PMC8694485

[B51] Watts C. R. Zhang Y. (2022). Progression of self-perceived speech and swallowing impairment in early stage Parkinson’s disease: Longitudinal analysis of the unified parkinson’s disease rating scale. *J. Speech Lang. Hear. Res.* 65, 146–158. 10.1044/2021_JSLHR-21-00216 34851686

